# The contribution of qualitative research in designing a complex intervention for secondary prevention of coronary heart disease in two different healthcare systems

**DOI:** 10.1186/1472-6963-6-90

**Published:** 2006-07-18

**Authors:** Mairead Corrrigan, Margaret E Cupples, Susan M Smith, Molly Byrne, Claire S Leathem, Pauline Clerkin, Andrew W Murphy

**Affiliations:** 1 Division of Medical Education, Queen's University Belfast, Northern Ireland, UK; 2 Division of Public Health and Primary Care, Queen's University Belfast, Northern Ireland, UK; 3 Department of Public Health and Primary Care, Trinity College Dublin, Ireland; 4 Department of Psychology, NUI Galway, Ireland; 5 Department of General Practice, NUI Galway, Ireland

## Abstract

**Background:**

Developing complex interventions for testing in randomised controlled trials is of increasing importance in healthcare planning. There is a need for careful design of interventions for secondary prevention of coronary heart disease (CHD). It has been suggested that integrating qualitative research in the development of a complex intervention may contribute to optimising its design but there is limited evidence of this in practice. This study aims to examine the contribution of qualitative research in developing a complex intervention to improve the provision and uptake of secondary prevention of CHD within primary care in two different healthcare systems.

**Methods:**

In four general practices, one rural and one urban, in Northern Ireland and the Republic of Ireland, patients with CHD were purposively selected. Four focus groups with patients (N = 23) and four with staff (N = 29) informed the development of the intervention by exploring how it could be tailored and integrated with current secondary prevention activities for CHD in the two healthcare settings. Following an exploratory trial the acceptability and feasibility of the intervention were discussed in four focus groups (17 patients) and 10 interviews (staff). The data were analysed using thematic analysis.

**Results:**

Integrating qualitative research into the development of the intervention provided depth of information about the varying impact, between the two healthcare systems, of different funding and administrative arrangements, on their provision of secondary prevention and identified similar barriers of time constraints, training needs and poor patient motivation. The findings also highlighted the importance to patients of stress management, the need for which had been underestimated by the researchers. The qualitative evaluation provided depth of detail not found in evaluation questionnaires. It highlighted how the intervention needed to be more practical by minimising administration, integrating role plays into behaviour change training, providing more practical information about stress management and removing self-monitoring of lifestyle change.

**Conclusion:**

Qualitative research is integral to developing the design detail of a complex intervention and tailoring its components to address individuals' needs in different healthcare systems. The findings highlight how qualitative research may be a valuable component of the preparation for complex interventions and their evaluation.

## Background

Coronary heart disease (CHD) morbidity and mortality in the Republic of Ireland (ROI) and the United Kingdom (UK) are among the highest in Europe [1,2]. Health service initiatives in both countries [1,3] have promoted secondary prevention and presented evidence for its effectiveness but its delivery remains sub-optimal [4,5]. The optimal mix of components of care remains uncertain [6]. The importance of randomised controlled trials of complex interventions is being recognised increasingly within healthcare planning [7].

Reviews of trials of disease management programmes for patients with established heart disease [6,8] have criticised their lack of detail and concluded that careful design and evaluation of different implementation models is needed. A framework for the development and evaluation of such programmes has been proposed by the Medical Research Council (MRC) [7]. This describes a phased approach which begins with a theoretical phase exploring the literature and progresses to a modelling phase which confirms the relevance of components identified from the literature, followed by an exploratory phase to refine the programme design before performing a randomised controlled trial.

The limited effectiveness of some previously reported programmes has been attributed to a failure to tailor the design of interventions to individual patients and practices [9,10]. A recent Cochrane review [11] recommended that future research should clarify how interventions address specific barriers to change in particular settings. Our understanding of how such barriers influence the delivery and uptake of healthcare interventions may be increased through qualitative research [7]. Integrating qualitative research in the design of an intervention may contribute to both optimising and evaluating it [12]. It has been hypothesised that it may also avoid an unsuccessful trial [13]. However, there has been little research on how qualitative methods are currently used in the context of randomised controlled trials of complex interventions and in improving the relevance of trial findings [14].

In this article we examine the contribution of qualitative research in the modelling and exploratory phases of developing an intervention for a randomised controlled trial for the provision of secondary prevention for CHD that addresses barriers identified by primary healthcare staff and patients in two different healthcare systems.

The study was approved by the research ethics committees of the Irish College of General Practitioners and Queen's University Belfast.

## Methods

### Setting

**Figure 1:**
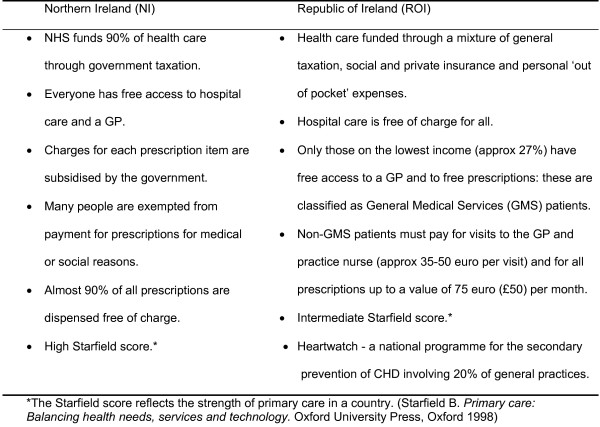
Characteristics of healthcare systems in Northern Ireland and Republic of Ireland.

This study relates to the modelling and exploratory phases of development of a complex intervention (the 'SPHERE' Study) which will be fully evaluated in an ongoing randomised controlled trial, the protocol for which has been published [15]. Four general practices were purposively selected to include one rural and one urban location in each of two healthcare systems – Northern Ireland (NI) and the Republic of Ireland (RoI). In the RoI both practices were also participating in a government initiative for the secondary prevention of CHD in general practice ('Heartwatch') [16]. Differences between the two healthcare systems are shown in Figure 1.

All participating practices were involved in undergraduate and postgraduate medical training and had previous experience of practice-based research. Further details about the general practices are found in Table 1. Limited time and resources restricted the study to these four general practices.

### Participant selection

All staff who carried out medical, nursing or administrative work in the practices and patients were invited to participate both in the modelling phase of data collection and in an evaluation of their experiences in the exploratory phase. Different patients were selected for the two phases of data collection in order to minimise the potential for bias and to facilitate access to a greater breadth of opinions regarding the intervention. The research nurse (CSL) liaised with staff to purposively select from within these practices, patients identified as having coronary heart disease (history of myocardial infarction, coronary artery bypass surgery or angioplasty). We did not include patients whose records indicated a diagnosis of angina because of difficulty confirming the validity of this diagnosis. Maximum variation sampling [17] was used to select male and female patients of varying age, diagnosis and length of time since diagnosis. Those with significant mental or physical illnesses likely to impair capacity to change lifestyle behaviour were excluded. Information about the study and an invitation to participate, a reply slip, and a questionnaire regarding aspects of lifestyle and health were posted to the selected patients. Non-responders were telephoned by the practice nurse two weeks later or approached opportunistically in the surgery.

### Data collection

Data from the modelling phase which explored the main issues to be addressed in developing an intervention were collected between March and June 2003. In each practice one focus group of staff and one of patients was held: in total, 29 staff members and 23 patients participated. Questions explored the barriers facing staff and patients in delivering and receiving secondary prevention and how the intervention would fit in with current activities. As it was only possible to carry out the focus groups during the day most of the patients (aged 48–74 years) who attended were retired from work. Their length of time since diagnosis varied from 3 months to 20 years. The staff focus groups were conducted during lunchtime in their own practice premises. (Attendee details are shown in Tables 2 and 3.)

**Figure 2:**
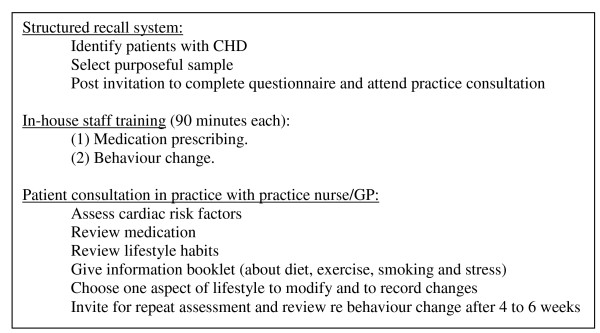
Exploratory pilot trial protocol.

Findings from the modelling phase were used to develop and tailor an intervention that addressed the barriers identified. The intervention was delivered in the exploratory phase, the details of which are shown in Figure 2. In total 27 patients participated; 20 attended for review (1 died) and 6 defaulted. Patients and staff who participated were invited to a focus group and an interview respectively to explore their experiences of the intervention. One patient focus group was held in each practice (March 2004): 17 patients participated, (aged 49–80 years). Their length of time since diagnosis varied from 4 months to 23 years (Table 3). In each practice a GP, nurse and practice manager were interviewed except in one practice where the nurse had left and in another where the practice manager was not available for interview (N = 10). Questions focused on the experiences of staff and patients in delivering and receiving the intervention respectively; effects of the intervention on motivation and lifestyle changes; usefulness of the information booklet and value of the staff training.

Data were collected in focus groups because of the strength of focus groups for generating new ideas through group interaction [18] and for facilitating access to the diverse opinions of a number of patients in a short space of time. Semi-structured interviews were used because only a small number of staff in each practice were involved in administering the intervention in the exploratory phase and interviews, rather than focus groups, facilitated detailed descriptions by these staff of their consultations with patients. The focus groups and interviews were divided between two experienced non-clinical qualitative researchers (MC and PC) who facilitated them. Although an interview schedule was used to promote standardisation of questioning, its semi-structured nature allowed the researchers flexibility to follow up issues that emerged during the focus groups and interviews. All were tape-recorded with the participants' consent. CSL and MB made written notes of interviews and observations of focus group interactions. CSL, who liaised with staff to facilitate the smooth running of the study, also made contemporaneous written records of her observations of the administration of the practices and individual discussions with staff in relation to their needs and experiences of the intervention. Investigator triangulation is recognised as one method of strengthening the validity and credibility of qualitative findings [17].

### Data analysis

Data analysis was conducted using the qualitative computer software programme NUDIST (N6). MC and PC initially analysed the transcripts separately to identify the main issues. These were indexed and categorised following discussions between the two researchers using a thematic analysis framework [19]. The analytical framework was applied to all the data and influenced the questions asked in subsequent interviews. Issues recorded by CSL in relation to individual discussions with staff were also categorised. The analytical themes under which to organise the data were then finalised following discussions between the two researchers and other members of the research team. Both researchers agreed that there were no new issues in the data collected from the fourth practice and that data saturation had been reached.

The data collection and analysis were influenced by the sociological theory of symbolic interactionism. This theory is based on three premises. Firstly, that human beings act towards things on the basis of the meanings that the things have for them. Secondly, that the meaning of such things arises from social interaction with others. Lastly, that these meanings result from a process of interpretation [20]. This theory led us to explore, for example, the social and cultural influences on patients' health beliefs and behaviours, and how staff related their relationship with patients to their motivation in pursuing secondary prevention with them.

## Results

### (1) Modelling phase

The main barriers facing staff and patients in delivering and receiving secondary prevention and how the intervention would fit in with their current activities were identified within the themes:

- Time and money

- Training

- Motivation

- Health beliefs

Most of the themes include quotations from staff and patients from both NI and the RoI that exemplify differences and similarities in their views and experiences as a result of their living in two different healthcare systems. The quotations were also chosen to include the diverse views and experiences of GPs, nursing and administrative staff.

#### Time and money

Practice staff in both healthcare systems identified lack of time and money as major barriers to delivering secondary prevention. However, RoI GPs complained more about financial insecurity. They relied for their income on a mixture of public and private finances (see Figure 1) and considered that the level of public funding for secondary prevention for GMS patients was inadequate. Staff also reported embarrassment in asking privately-funded non-GMS patients to pay for extra visits for preventive care and were conscious that some non-GMS patients perceived these visits as opportunities for them to make more money. It appeared that mechanisms of payment for services could influence decisions to invite patients for review and impact on the doctor-patient relationship.

*'Our GMS contract doesn't allow us to do prevention...if we raise the issue and do it properly the amount of time and effort...it's financially disastrous.' *(Practice 1, GP2)

*'The private patient thinks that you're bringing them back too often to make money out of them.' *(Practice 1, GP2)

*'It's kind of embarrassing when they go, "but sure I was told to come back for that".' *(Practice 1, practice manager)

Arrangements for payment also influenced patients' readiness to attend. Their perceptions of the value of preventive healthcare varied with some patients more willing to pay for preventive healthcare than others. Various comments indicated the balance that existed in decisions to uptake services. Healthcare costs were considered in competition with other living expenses with some patients disagreeing that preventive healthcare provided value for money.

*'Your health is your wealth.' *(Practice 1, female patient 3)

*'I would feel bad and I still wouldn't go to the doctor...I have a lot of little holes for the money.' *(Practice 1, male patient 2)

In contrast to these reports from the RoI, the influence of financial cost was not recognised by NI patients, all of whom received free prescriptions. Also, NI staff tended to disregard any financial loss associated with providing secondary prevention.

*'It's not particularly well recompensed...none of the extra clinics currently are in general practice... they cost a lot more to run than we actually get for them... it's just because we think that it's a good service to offer to our patients.' *(Practice 4, GP1)

#### Training

In both systems staff identified how updating their knowledge would improve their confidence in prescribing. They were aware of a need for training, specifically in relation to prescribing and behaviour change, which should include evidence to support their clinical activity.

*'specific training has to be given on the different issues like management of cholesterol...so that...you're...comfortable enough that you change what has been done in the hospital' *(Practice 1, GP1)

*'They haven't tried so why should we be wasting our energy on this person?' *(Practice 1, GP2)

*'For sometimes you can get a wee bit frustrated...if people don't come in you think...what's the point of this?' *(Practice 4, practice nurse)

Lack of time and money led staff to emphasize that training should be short, focused and integrated into practice timetables. Attending training sessions outside of practice premises or lasting more than two hours were not acceptable options.

*'It has to be very relevant to what you want to do...I think you're going to have to be very focused' *(Practice 4, GP2)

*'Getting down to the basics awful quickly...everything in the shortest possible time...I would not go for a 2 day course...2 hours' *(Practice 1, GP2)

#### Motivation

Staff considered that poor motivation to comply with secondary preventive advice was reflected in patients' non-attendance at appointments. They attributed it particularly to those who lived in socio-economically deprived areas in both healthcare systems. However, they also identified that patients in employment had difficulties attending during surgery hours because of financial implications of taking time off work and they expressed a reluctance to ask patients to take time off work to attend appointments.

*'There are people who genuinely feel that they can't afford to take time off work to come to us during our surgery time.' *(Practice 3, practice nurse)

*'I'm very conscious of asking them to take time off work' *(Practice 2, practice nurse)

Recognition of these influences led to staff tailoring their services for patients. However, they also recognised the possibility of creating a culture of dependency, characterised by RoI staff to be more common among GMS than non-GMS patients. The inter-play of State funding of healthcare and personal responsibility was considered to influence both the provision and uptake of care.

*'Give them the information and show them how to manage things themselves... you'll get away with that with the private patient...whereas the GMS patient isn't used to doing that because he likes somebody to take care of everything for him.' *(Practice 1, GP1)

#### Health beliefs

Patients, particularly those living in socio-economically deprived urban areas, in both systems, emphasised their beliefs relating to the role of stress in causing their heart condition. The perception of a behaviour change having possible adverse effects on their stress levels influenced their compliance with advice.

*'Smoking did contribute to it but it wasn't the main factor, no way, it was stress...doing away with the stress means an awful lot more to me than doing away with those two cigarettes.' *(Practice 2, female patient 1)

*'..that half hour with a glass of sherry and a cigarette and the crossword is as important...as going for a walk.' *(Practice 1, female patient 4)

They reported internal stress in knowing that they should take exercise but feeling physically unable to do so. Some feared their heart condition would deteriorate if they exercised and criticised how current service provision did not deal adequately with reducing their stress or tackling their fears.

*'I was told to exercise and start walking...I couldn't do it, I was absolutely shattered so that was making me more stressful.' *(Practice 3, female patient 2)

*'...more important than your body is your...state of mind...there's not enough dealing with what goes on in your head.' *(Practice 2, female patient 2)

### (2) Exploratory phase

The findings were used in tailoring the intervention to address the barriers, described above, to make it applicable to each particular setting within both healthcare systems. The experiences of staff and patients in delivering and receiving this intervention, its perceived effects on motivation and lifestyle changes, the usefulness of the patient information booklet and the value of the training delivered were explored. Their experiences of the intervention are presented within the same themes as those already identified as barriers to the uptake and delivery of secondary prevention.

#### Time and money

All staff members approved of the financial recognition provided for practices in both systems and that no patients were charged for visits. In respect of the differing funding arrangements in NI and RoI patient reviews were integrated differently into existing programmes. In NI nurses, doctors and receptionists liaised within each practice to ensure that patients were not called within short time intervals to attend different clinics where similar assessments were made. In the RoI integration with Heartwatch aimed to minimize additional practice administration. However, this proved unsuccessful as staff and patients were unclear about the difference between Heartwatch and the intervention.

*'There is a lot of cross-over between the two...we already seemed to be doing a lot of it anyway.' *(Practice 1, GP)

In all but one of the practices administration of the intervention was delegated to the practice nurses. With the exception of one practice nurse who had been allocated protected time for the study, they all found administration of the intervention time-consuming because of their busy workloads. Despite efforts to minimize it, they resented the extra time they had to spend on record-keeping and paperwork.

*'... it is good. It's just the initial workload.' *(Practice 3, practice nurse)

#### Training

Staff criticized the behaviour change training for being too theoretical and not giving enough emphasis to the practicalities of implementing it. They recommended that opportunities to watch a consultation and to practise what they learned in 'role play', should be included.

*'... it would have been quite useful...for me to actually watch a consultation...that might have made it more hands on and then I would have known I was doing it right.' *(Practice 2, practice nurse)

*'Just to make that jump from theory to practice.' *(Practice 1, GP 1)

Staff responded positively to the medication training; they appreciated opportunities for case-based learning.

*'that particular group thing...... was very good.' *(Practice 2, practice nurse)

#### Motivation

The intervention was designed to tailor consultations to patients' socio-economic circumstances and health beliefs. Staff reported that in doing so they became more knowledgeable about their patients and were able to personalise lifestyle advice. They were encouraged by patients' positive responses to advice but found difficulty motivating 'model patients' who had already 'healthy' lifestyles and older people who felt it was too late in life to make significant changes.

*'...saw some of the positive effects of it .........really personalising it and looking at where they are and where they're coming from.' *(Practice 4, practice nurse)

*'...... model patients. So that was one of the issues, trying to identify an area for them to work on.' *(Practice 1, GP)

Patients valued the information given in the booklet about heart disease, lifestyle change and medication. However, they were reluctant to complete the self-monitoring pages within it, relating to lifestyle change: they considered this was unhelpful.

*'I know in my head what I have to do, so I don't need to run to the book all the time.' *(Practice 4, male patient 1)

*'...trying to fill that smoking bit in everyday would put the cigarettes in my mind.' *(Practice 3, female patient 2)

Patients found it difficult to identify specific goals in relation to lifestyle behaviours, particularly stress. Staff also questioned the practicalities of setting goals in relation to stress management.

*'It's one of those things you don't see or you can't hear or feel, you can't tell a doctor or anybody how stressed you are...it's a very personal thing, you can't describe it and another person can't relate to how you feel.' *(Practice 2, male patient 1)

*'I just don't know how practical it is, I mean a stress self-monitoring form, yeah, I'd be interested to see how many people actually do fill that in.' *(Practice 1, GP)

Some staff used the booklet in consultations as a prompt to remind them of relevant issues but others felt that it obstructed the flow of individual consultations. They cautioned against relying on it for recording patients' progress since many failed to bring it to review consultations.

### Application of findings to intervention design

The implications of financial costs for both staff and patients in the RoI identified in the modelling phase had not been reported previously and it was important to address these within the development of a structured system to promote arrangement of and attendance at review appointments. In keeping with previous studies [12,21,22] training needs and time constraints identified by staff informed design of a training programme (similar for both systems) and administrative elements of the intervention (different details for different systems and practices). Patients' reports of the importance of stress within their health beliefs were addressed in an information booklet.

The qualitative data from the exploratory phase led to the intervention being adapted for the main trial. Although we had considered, within the exploratory phase, that we had minimised administrative elements of the intervention, resentment among staff of the time required for administration resulted in the paperwork and overall administrative load being reduced. The confusion caused by integrating the intervention with the initiative Heartwatch (RoI) [16] led to the decision to exclude Heartwatch practices. Flexibility in tailoring structured recall programmes to practices' needs was increased. Patient information was amended to increase examples of goal setting for behaviour change and omit self-monitoring records. The qualitative research highlighted the need for the training and the approach to behaviour change to be considered within specified theoretical frameworks which addressed aspects of patient and staff motivation, and patient health beliefs using practical examples of application of theory and case-based learning. A full discussion of the behaviour change theories relevant to the intervention has already been reported [23].

## Discussion

This study found that qualitative research methods make significant contributions to the development of a complex intervention and in testing its feasibility and acceptability to staff and patients in two different health care systems. Integrating the qualitative research helped to identify clearly how barriers influenced the provision and uptake of secondary prevention and how components of the intervention could be tailored to meet individual needs in different settings. Using similar qualitative methods to explore experiences of the intervention allowed clarification of how specific components required further tailoring to prevent a randomised controlled trial of an intervention which was unlikely to be successful.

The relevance of seeking information about the extent to which a theory-based intervention can be applied appropriately in a particular setting has been reported previously within the context of an exploratory trial [12] and hypothesised in relation to a trial of an unsuccessful intervention [13]. We sought to expand on this body of literature by conducting, within the modelling phase of development of the intervention, a qualitative exploration of aspects of organisational arrangements and of perspectives of staff and patients in the setting in which a randomised controlled trial was to be delivered. Qualitative findings from the exploratory phase informed further the intervention design. Our initial findings clarified the relevance of key components of effective interventions identified in the literature [9,12,24-27] within the context of two different healthcare systems.

### Standardisation and tailoring

The findings facilitated the description of the constant and variable aspects of components of the intervention [7] and definition of its standardisation in terms of 'form and function' [28]. The information indicated how the desired function of intervention components (structured reviews, practitioner training and patient information) could be fulfilled whilst tailoring their form of delivery to the needs of different practices, practitioners and patients. Detailed examples of how the information influenced the development of the intervention [29] and how it was applied specifically to the delivery of behaviour change training [23] have been described previously. The inclusion of stress in the patient information booklet highlighted the importance of incorporating patients' perspectives. We also conducted objective staff evaluations of training delivered in the intervention through questionnaires relating to the location, duration, appropriateness and clarity of content of training. These did not reveal the depth of information gathered through this qualitative work, for example how the training could be made more relevant to particular practitioners, practices and patient needs; background information about staff in respect of ability and training received; and detailed information about organisational difficulties and staff-patient relations. This reinforces the strengths of qualitative research methods in being able to "reach the parts other methods cannot reach" [30].

### Strengths and limitations

This is a small study which reflects the tension that often exists between conducting research that is rigorous and the practical constraints of time and funding. To overcome these limitations the practices were chosen to include small and large practices in rural and urban locations to reflect the diversity of practices in both healthcare systems. The validity of the findings is supported by reports of similar barriers identified in other settings [10,12,21,22]. However, the inclusion of only four practices may have restricted the breadth of information which would be of relevance to the development of the intervention and trial design. Moreover the fact that they were all teaching practices might suggest that the findings represent the "best case scenario" for delivery of secondary prevention. Therefore it is important to place these findings within the context from which they were derived. However there was consensus amongst the research study team that data saturation had been achieved as no new issues had emerged from the interviews in the fourth practice.

The amount and type of data that were collected may also have been influenced by how well the researchers interacted with the respondents during the interviews that may reflect their different interpersonal skills. However, similarities in the respondents' responses recorded by the different researchers indicate that the data were not adversely affected by their personal characteristics.

Using 'pre-existing groups' for staff focus groups promotes 'naturally occurring' data, with group discussions reflecting participants' shared work experiences. However hierarchical relations within the group may censor some participants [18] and observations which appeared to confirm this justified our use of individual interviews in the exploratory phase. Analysis of the focus group transcripts confirmed the researchers' observations that discussions tended to be dominated by the GPs, nurse practitioners or practice nurses and practice managers while the treatment room nurses and receptionists were more silent. However, separate informal discussions between CSL and practice staff revealed no new issues.

A particular strength of the study would be its indication of the differences in attitudes engendered in staff and patients by different healthcare systems. The study took place prior to implementation of the 2004 GP contract in NI. The changed arrangements for payment for management of CHD may impact on the intervention in the main trial but the current findings and awareness of the impact of background policy should assist in our interpretation of the findings. The implications of costs for practices and patients will be taken account of in the main trial.

## Conclusion

We have shown that qualitative findings in the development of a complex intervention contribute to refinement of the design detail by identifying and addressing barriers and facilitators to implementing the intervention in the main trial in a way that is acceptable to staff and patients [31]. Our study also indicates the importance of taking cognisance of background health service policy and of the economic circumstances of both general practices and patients, in trials of health services interventions. The findings highlight how qualitative research may be a valuable component of the preparation for complex interventions and their evaluation.

## Competing interests

The author(s) declare that they have no competing interests.

## Authors' contributions

MC designed the study, collected and analysed the data and drafted the manuscript. MEC, SMS, MB and AWM designed the study, delivered aspects of it, analysed data and drafted the manuscript. CSL collected and analysed data and delivered aspects of the study. PC collected and analysed data. All authors read and approved the final manuscript.

## Pre-publication history

The pre-publication history for this paper can be accessed here:



## Figures and Tables

**Table 1:** Characteristics of practices

**Practices**	**No. of GPs**	**No. of nurses**	**No. of admin staff**	**Patient list size**	**CHD register**
Practice 1 (RoI)	4.5	1	3	GMS 1600	under construction
Practice 2 (RoI)	6	2	3.5	GMS 3000	under construction
Practice 3 (NI)	5	3	7	8500	yes
Practice 4 (NI)	5	2 (part-time)	3	4500	yes

**Table 2:** Staff focus group participants: numbers in different staff categories in each practice who attended during the modelling phase

Staff category	Practice 1 (RoI)	Practice 2 (RoI)	Practice 3 (NI)	Practice 4 (NI)	Totals
GP	4	3	2	2	11
Nurse	1	2	3	1	7
Admin. (Reception & manager)	3	3	3	2	11
Total staff	8	8	8	5	29

**Table 3:** Characteristics of patient focus group participants during the modelling and exploratory phases

Modelling Phase					
	Practice 1 (RoI)	Practice 2 (RoI)	Practice 3 (NI)	Practice 4 (NI)	Total

Male	3	8	2	4	17
Female	1	2	1	2	6
Total (total invited)	4 (5)	10 (20)	3 (15)	6 (10)	23 (50)

Exploratory Phase					

Male	2	2	3	4	11
Female	1	2	2	1	6
Total (total invited)	3 (5)	4 (6)	5 (8)	5 (8)	17 (27)
